# Advances in Crop Breeding Through Precision Genome Editing

**DOI:** 10.3389/fgene.2022.880195

**Published:** 2022-07-14

**Authors:** Gauri Nerkar, Suman Devarumath, Madhavi Purankar, Atul Kumar, R. Valarmathi, Rachayya Devarumath, C. Appunu

**Affiliations:** ^1^ Molecular Biology and Genetic Engineering Laboratory, Vasantdada Sugar Institute, Pune, India; ^2^ Vidya Pratishthan’s College of Agricultural Biotechnology, Baramati, India; ^3^ ICAR-Sugarcane Breeding Institute, Coimbatore, India

**Keywords:** Genome editing, crop breeding, new breeding techniques, CRISPR, disease resistance, abiotic stress tolerance, biofortification, climate-resilient crops

## Abstract

The global climate change and unfavourable abiotic and biotic factors are limiting agricultural productivity and therefore intensifying the challenges for crop scientists to meet the rising demand for global food supply. The introduction of applied genetics to agriculture through plant breeding facilitated the development of hybrid varieties with improved crop productivity. However, the development of new varieties with the existing gene pools poses a challenge for crop breeders. Genetic engineering holds the potential to broaden genetic diversity by the introduction of new genes into crops. But the random insertion of foreign DNA into the plant’s nuclear genome often leads to transgene silencing. Recent advances in the field of plant breeding include the development of a new breeding technique called genome editing. Genome editing technologies have emerged as powerful tools to precisely modify the crop genomes at specific sites in the genome, which has been the longstanding goal of plant breeders. The precise modification of the target genome, the absence of foreign DNA in the genome-edited plants, and the faster and cheaper method of genome modification are the remarkable features of the genome-editing technology that have resulted in its widespread application in crop breeding in less than a decade. This review focuses on the advances in crop breeding through precision genome editing. This review includes: an overview of the different breeding approaches for crop improvement; genome editing tools and their mechanism of action and application of the most widely used genome editing technology, CRISPR/Cas9, for crop improvement especially for agronomic traits such as disease resistance, abiotic stress tolerance, herbicide tolerance, yield and quality improvement, reduction of anti-nutrients, and improved shelf life; and an update on the regulatory approval of the genome-edited crops. This review also throws a light on development of high-yielding climate-resilient crops through precision genome editing.

## Introduction

The global climate change has a direct impact on the food security, agriculture, crop production and plant health (Tirado et al., 2010). According to the world population data sheet 2020, the world population is projected to increase from 7.8 billion in 2020 to 9.9 billion by 2050. Global cropland area per capita has decreased continuously from about 0.45 ha per capita in 1961 to 0.21 ha per capita in 2016 (https://www.fao.org). Further, the available area for cultivation is degraded due to various factors such as loss of agricultural land to non-agricultural uses, intensive use of land for cultivation through multiple cropping, reduction in fallow periods, excessive use of agrochemicals, and spread of monocultures (Alexandratos and Bruinsma, 2012). These factors undermine the long-term productive potential of the available cultivable land. Thus, on one hand when the global demand for food is increasing, there is a decline in the availability of the cultivable land for the production of food crops. Table 1 lists the important crops for food security and comparison of their global production during 2016–17 and 2021–22 (data accessed from: http://www.fao.org/faostat/en/#home). This data show that the harvested area for the most grown crops of the world has remained fairly constant over the past 5 years and there is also a slight increase in the yield and production of these crops. Increasing the crop productivity in the face of the global climate change is therefore most important challenge in front of the crop breeders. The other important factors that limit the agricultural productivity are limited water availability and irrigation, declining soil fertility, untimely rainfall, high temperature, pests and pathogens (Singh et al., 2020).

**TABLE 1:** Global production of top 10 crops essential for food security ranked based on their global harvested area.

Rank based on Harvested area	Crop	Harvested area (million hectares)		Production (million metric tons)		Yield (metric tons per hectare)	
		2016–17	2021–22	2016–17	2021–22	2016–17	2021–22
1	Wheat	222.11	222.11	753.09	778.6	3.39	3.51
2	Maize	183.06	203.89	1,065.11	1,210.45	5.82	5.94
3	Rice	161.48	166.47	481.54	513.03	4.45	4.60
4	Soybean	121.11	130.10	348.04	350.72	2.87	2.70
5	Barley	48.21	48.48	147.04	145.10	3.05	2.99
6	Sorghum	41.82	41.75	63.18	65.59	1.51	1.57
7	Rapeseed	33.65	37.73	68.86	71.18	2.05	1.89
8	Cottonseed	28.62	31.52	38.70	43.47	1.35	1.38
9	Cotton	29.44	32.07	105.88	120.20	783	816
10	Peanut	26.15	29.65	42.34	50.60	1.66	1.71

The current efforts are focused on increasing the crop productivity without using pesticides and fertilizers. Conventional breeding programs are often laborious, time-consuming and difficult. Genetic engineering has greatly simplified the process of development of novel and improved varieties with better agronomic traits like disease resistance, abiotic stress tolerance, a better shelf life as well as improved crop productivity (Nerkar G. et al., 2018). The recent emergence of the novel plant breeding technologies like genome editing has opened up new doors for precise modification of the plant genomes without the introduction of foreign DNA (Altpeter et al., 2016). Their successful application in the development of elite germplasm, with high yield, quality, and resistance against biotic and abiotic stresses appears promising (Fiaz et al., 2020). Therefore, there is an urgent need to utilize these technologies for the development of novel and improved crop varieties to overcome the difficulties faced in the conventional breeding programs (Fiaz et al., 2021a).

Genome editing can facilitate genome modification by creating precise changes at specific sites of the genome and the reagents used in this process can be delivered into the cell without incorporating DNA into the genome (Zhang et al., 2016; Bandyopadhyay et al., 2020; Ma et al., 2020). The mutations resulting from genome editing are similar to those occurring in nature which potentially simplifies their regulation, unlike the traditional GMO crops. Another remarkable feature of this technology is that it creates inheritable mutations in the genome with a low probability of generating off-targets. Genome editing creates DNA modifications such as deletions, insertions, single nucleotide substitution (SNPs), and large fragment substitution. The site-specific nucleases (SSNs) that bring about nucleotide excision are: engineered homing endonucleases or mega-nucleases (MNs) (Smith et al., 2006), Zinc-Finger Nucleases (Kim et al., 1996), transcription activator like effector nucleases (TALENs) (Christian et al., 2010), and CRISPR-associated protein (Cas) (Jinek et al., 2012).

**FIGURE 1:**
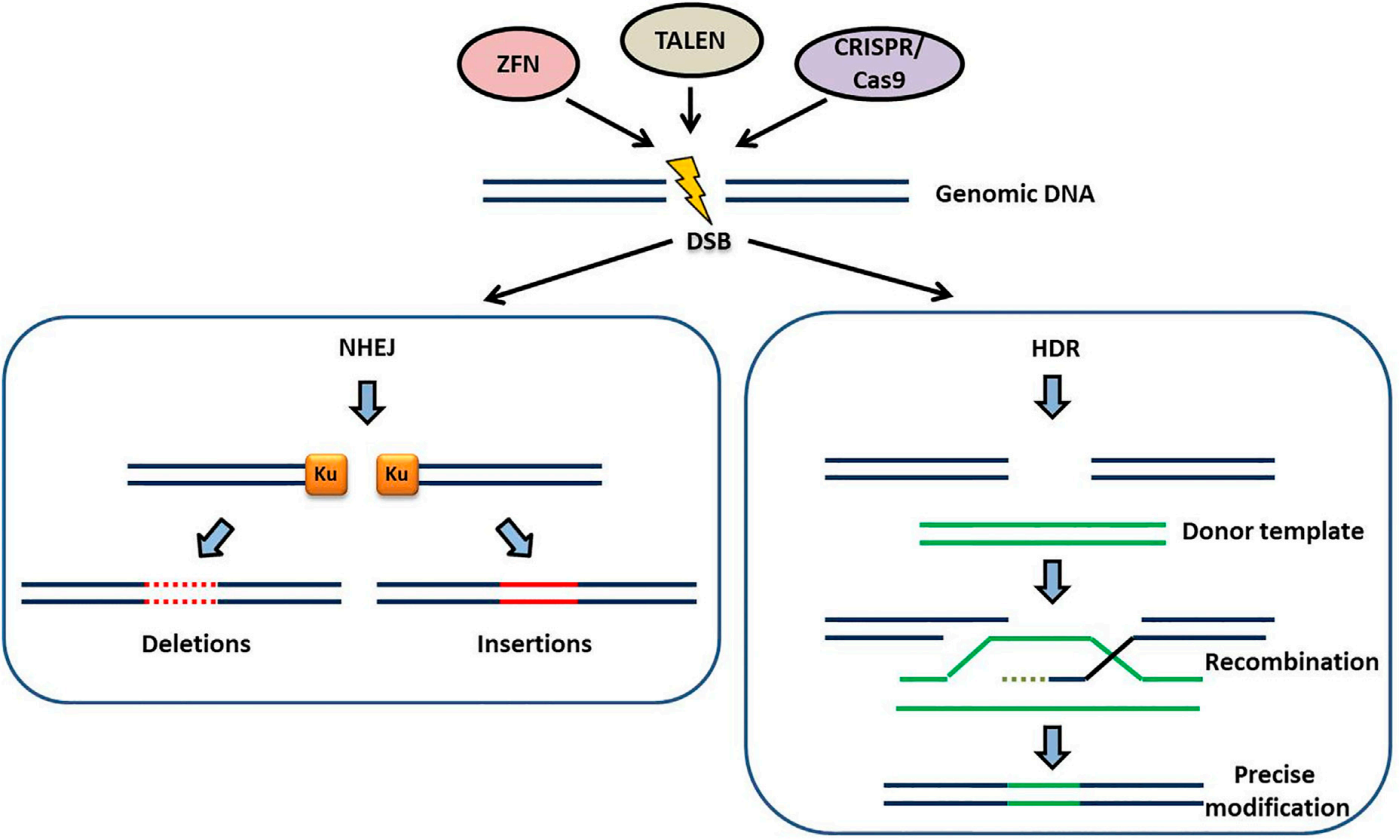
The two major DNA repair mechanisms for repairing the double-stranded breaks (DSB) generated by SSNs (ZFN, TALEN, CRISPR/Cas9): In the absence of a donor template the DSB repair occurs by the non-homologous end joining (NHEJ) pathway. Ku, a dimeric protein complex binds to the DNA DSBs and heals broken ends of chromosomes generating small deletions (dotted *red lines*) and/or insertions (*continuous red lines*). This process is erroneous and can generate indels of variable length at the target site. In the presence of a donor template, homologous to the site of DSB, DSB repair occurs by homology-directed repair (HDR) pathway. HDR is less error prone compared to NHEJ and ensures precise modifications at the target site through recombination of the target locus with the donor template.

The site specific nucleases create double-stranded breaks (DSBs) in the genome. The era of precise genome editing in plants began with the discovery of I–SceI induced DSBs that enhanced homologous recombination in plants (Puchta et al., 1993). SSNs are programmed to recognize the preselected genomic sites and they make use of cellular DSB repair mechanisms such as non-homologous end joining (NHEJ) or homology-directed repair (HDR) (Figure 1). In NHEJ, a gene is rendered non-functional by random insertion or deletion of DNA at the cut site before reattaching of the free DNA ends (Puchta 2005). HDR involves addition of a Donor DNA of choice which is homologous to the site of the break. Cells use this as a patch to repair the DNA (Haber 2000) (Figure 1). Using the HDR pathway, scientists can introduce a new gene with vital function or correct a mutation by replacing the mutated sequence with a healthy sequence (Symington and Gautier 2011).

The global food security is largely affected by the changing climatic conditions, significant yield gaps between the actual yield and the potential yield, decrease in the number of farmers, lack of transportation infrastructure, post-harvest losses due to low shelf life of crops (Mackelprang and Lemaux, 2020; Fiaz and Wang, 2021c). Precision genome editing can help in addressing these problems by generating plants with sufficient yields in spite of changing climatic conditions. The crop varieties which remain underutilized due to low yields, high disease susceptibility can be made more resilient using genome editing. They can make specific plants a source of essential nutrients that are lacking in the diets of some populations.

Crop breeding has been greatly accelerated after the introduction of genome editing tools. Therefore, there is an urgent need to review the advances in crop breeding through precision genome editing. Here, we provide an overview of the different breeding approaches for crop improvement; genome editing tools and approaches used for crop improvement; agronomic traits such as disease resistance, abiotic stress tolerance, herbicide tolerance, yield and quality improvement, reduction of anti-nutrients, improved shelf life and an update on the regulatory approval of the genome-edited crops.

## Mechanism of Action of Different Genome Editing Technologies Used for Crop Improvement

Meganucleases have the target sites of up to 18 bp (Smith et al., 2006). ZFNs have a non-specific *Fok*I endonuclease domain combining with multiple zinc-finger DNA-binding domains that recognize a 3 bp module (Kim et al., 1996). TALENs consist of a *Fok*I endonuclease domain which pairs with multiple transcription activator-like effector domains that recognize single base pairs (Christian et al., 2010). TALENs have been widely applied in genome editing of crops, owing to their higher target binding specificity and generation of lesser number of off-targets compared with ZFNs (Liu et al., 2021).

In bacteria and archaea, Clustered Regularly Interspaced Palindromic Repeats (CRISPR) in combination with Cas (CRISPR associated) proteins form an adaptive immune system (Mohanraju et al., 2016). CRISPR-Cas immune systems consist of three distinct stages *viz.* adaptation, during which the short DNA fragments (spacers) from invading viruses and plasmids are recognized and acquired, processed and integrated into the CRISPR locus (Jackson et al., 2017); transcription during which the transcription of CRISPR locus to a long pre-CRISPR RNA (pre-crRNA) and the maturation of pre-crRNA to crRNA (guide RNA) occur (Charpentier et al., 2015); and finally interference in which the complementary target DNA sequences are recognized by Cas effector nucleases using the guide RNA. Consequent to the recognition of the target DNA, Cas effectors bind to the target DNA and generate a double-stranded DNA break (DSB) (Jinek et al., 2012).

There are six primary types of the CRISPR-Cas systems. Types I, III, and IV are characterized by multi-subunit effector complexes, while types II, V, and VI consist of single-subunit effector (Koonin et al., 2017; Shmakov et al., 2017). The class 2, type II clustered regularly interspaced short palindromic repeat (CRISPR)/Cas9 (CRISPR-associated) system from *Streptococcus pyogenes* is the ground-breaking technology for genome editing discovered around a decade ago, which is based on RNA-guided engineered nucleases (Jinek et al., 2012). While Meganucleases, ZFNs and TALENs recognize their sequence targets through protein/DNA interactions, CRISPR/Cas9 achieves targeting through a guide RNA (sgRNA). sgRNAs are short nucleotide sequences (∼20 nt) with a specific sequence that can target the genomic sequence of interest. The Cas9 nuclease then cleaves the resulting RNA/DNA complex. Consequently, a DSB is created at the target site containing a conserved protospacer adjacent motif (PAM). The repair occurs by NHEJ which creating indels in the protein-coding regions causing frameshift or knock-down of the desired genes (Basso et al., 2020). The simplicity of DNA targeting through base-pairing has led to the quick and broad adoption of CRIPSR/Cas9 reagents for genome editing (Ahmar et al., 2021; Liu et al., 2021). The most widely used Cas9 is derived from *Streptococcus pyogenes* (SpCas9) which requires a protospacer adjacent motif (PAM) sequence of “NGG” in the target DNA sequence. Other Cas9 variants differing in their PAM requirements (SpCas9-VQR-“NGA”, SpCas9-EQR- “NGAG”, Cas9-NG-“NG”, and xCas9 3.7-“NG/GAA/GAT”) have also been used for plant genome editing (Anzalone et al., 2019; Zhang et al., 2019; Nadakuduti and Enciso-Rodríguez, 2021).

Cas12 nucleases belonging to class 2, type-V CRISPR systems were later added to the CRISPR toolbox (Zetsche et al., 2015). The major differences between Cas9 and Cas12 nucleases are that Cas12 nucleases are mostly guided by a single crRNA of ∼42 nt compared to the Cas9 guide RNA of ∼100 nt; unlike Cas9, Cas12 effectors lack HNH domain and possess only RuvC-like domain that cleaves both strands of the DNA target site resulting into a staggered cut with a 4–5 nt 5′ overhang (Zetsche et al., 2015). LbCas12a is the most widely used Cas12 variant for gene-editing in plants and it recognizes a T-rich PAM “TTTV” (Zhang et al., 2019). Engineered variants of Cas12a with increased activities and target ranges have also been developed (Kleinstiver et al., 2015). A distinct feature of Cas12a is that it does not require tracrRNA for the processing of mature crRNA making it advantageous for multiplex gene editing, transcription, epigenetic modulations and base editing (Safari et al., 2019).

The most recent addition to the CRISPR toolbox is the CRISPR-Cas8, which is a hypercompact type-V CRISPR system consisting of a single Cas8 protein of ∼70-kDa that is about half the size of Cas9 or Cas12a (Nadakuduti and Enciso-Rodríguez, 2021). Like Cas12a, Cas8 also does not require a tracrRNA and generates a staggered cut with 5′-overhangs (Pausch et al., 2020) and requires the PAM of 5′-TBN-3′ (where B = G, T, or C). CRISPR-Cas8 mediated genome editing has been reported in Arabidopsis with an editing efficiency of 0.85% (Pausch et al., 2020).

Recently, precise modification of DNA and RNA has also been reported at single-base level through base editing which can convert one target DNA nucleotide to another. Base editors (BEs) can precisely modify nuclear and organellar genomes (DNA BEs) as well as transcriptomes (RNA BEs) of dividing as well as non-dividing cells (Molla et al., 2021). BEs consist of a catalytically impaired Cas nuclease (dCas9: D10A and H840A) that is fused to a nucleotide deaminase and DNA repair proteins. Unlike the SpCas9-generated DSBs that are repaired by error prone NHEJ, the BE-generated individual nicks are repaired by a more precise base excision repair pathway (BER), thus, minimizing the undesired by-products due to gene-editing (Ran et al., 2013). DNA BEs can be classified as: cytosine BEs (CBEs), adenine BEs (ABEs), C-to-G BEs (CGBEs), dual-base editors and organellar BEs. Genome editing using CBEs has been reported in some of the major crops like Arabidopsis (Chen et al., 2017), rice (Shimatani et al., 2017), wheat (Zhang et al., 2019), maize (Zong et al., 2017), tomato (Hunziker et al., 2020), potato (Veillet et al., 2019a), cotton (Qin et al., 2020), soybean (Cai et al., 2020), and rapeseed (Cheng et al., 2021). Although base editors can bring about base transitions without DNA donors, they cannot be used with other base transversions, insertions or deletions.

The ‘search and replace’ prime editing (PE) is the most recent and by far the most advanced tool for genome editing which can copy the desired edit incorporated within the guide RNA without using DSBs or donor repair template (Anzalone et al., 2019) and generate targeted insertions or deletions, or directly install precise transition and transversion mutations at targeted genomic loci.

Prime editing is mediated by a complex consisting of prime editing guide RNA (pegRNA) and a catalytically impaired Cas9 endonuclease [nCas9 (H840A)] that is fused with a reverse transcriptase from engineered moloney murine leukaemia virus (M-MLV RT). This complex binds to the regions of protospacer and PAM. nCas9 (H840A) nicks the edited sequence by RuvC-like domains at a position three base pairs upstream of PAM. Subsequently, as the primer binding site (PBS) matches to the exposed 3′ end of the edited sequence, reverse transcription is initiated whereby the editing information is transformed to the edited sequence from the RT template. A mismatch formed in the heteroduplex DNA which contains one edited and one unedited sequence is repaired by using the edited sequence as a template (Hao et al., 2021). Prime editing has been reported in cereal crops (Butt et al., 2020; Lin et al., 2020; Tang et al., 2020; Fiaz et al., 2021b) and has an advantage of fewer bystander mutations compared to base editing and also less restricted to PAM availability compared to the other genome editing methods (Anzalone et al., 2019). However, base editors still remain widely applicable due to their improved efficiency with superior on-target and off-target DNA editing profiles, product purity, and DNA specificity (Anzalone et al., 2019; Yu et al., 2020). Thus, the choice of suitable editing strategy largely depends on the specific application such as the desired edit, availability of PAMs, editing efficiency and generation of off-target/bystander mutations (Hao et al., 2021).

## Breeding Approaches for Crop Improvement

The traditional breeding approaches have greatly contributed to genetic improvement of the present day elite crop varieties. The recent advances in the traditional breeding methods include wide crosses, introgression from wild-crop by hybrid breeding, mutation breeding, double haploid technology, tissue culture-based approaches like embryo and ovule rescue and protoplast fusion. Introgression through hybridization and back-crossing is one of the most widely adopted methods for developing elite crop varieties with desirable traits. It is also an important breeding strategy for transferring desirable traits from wild species to cultivated varieties. Interspecific gene flow has contributed to the origin of crop plants, restoration of crop diversity after domestication and to the adaptation to challenging environments (Foria et al., 2022). Introgressive hybridization has been used to tap the secondary gene pool for accessing the genetic variation for crop improvement in wheat (Mujeeb-Kazi and Asiedu, 1990), rice (Jena, 2010), potato (Jansky and Oeloquin, 2006), tomato (Schouten et al., 2019), cassava (Wolfe et al., 2019). Wild-crop introgressive hybridization has been used for incorporating disease resistance traits into newly released crop varieties (Hajjar and Hodgkin., 2007). Another frequently used approach for crop breeding is inbreeding. Inbred varieties are produced by self-fertilization in order to preserve the original traits and to produce true breeding cultivars which can be as parents in the hybridization programmes. Inbreeding can be used to improve the results of selection when the heritability for a trait is small. In hybrid breeding, two different inbred varieties are crossed to produce an offspring with stable characteristics and hybrid vigor, where the offspring is much more productive than either parent (Caligari, 2001). Hybrid crop varieties perform better than their inbred progenitors in an array of crops like maize and oil palm (Labroo et al., 2021).

In mutation breeding, genotypes showing spontaneous mutations are selected for breeding or mutations are induced using physical or chemical mutagens to create mutant phenotypes with desired traits (Devarumath et al., 2015; Purankar et al., 2022). Marker assisted breeding makes use of molecular markers, in combination with linkage maps and genomics, to alter and improve the crop traits on the basis of genotypic assays (Jiang, 2013). The morphological (trait-specific), proteinaceous (isoenzyme), cytological (chromosome-specific), and DNA markers have been used in plant breeding. DNA markers have been extensively utilized for marker-assisted selection of crop plants (Madina et al., 2013; Kumawat et al., 2020). Recently, the advanced molecular breeding tools such as SSRs, Indels, SNPs, genome sequencing, genotype by sequencing, and microRNAs have been used for crop improvement (Devarumath et al., 2014; Platten et al., 2019; Bohar et al., 2020) to confer biotic and abiotic stress tolerance (Shriram et al., 2016; Devarumath et al., 2019).

Genetic engineering of plants commenced nearly three decades ago after the first successful regeneration of a transgenic plant was reported from transformed cells of tobacco (Barton and Brill, 1983) petunia, and tomato (Horsch et al., 1985). The invention of biolistic gene gun (Sanford et al., 1987; Klien et al., 1992) paved a way into transformation of recalcitrant crops which were not amenable to *Agrobacterium-*mediated transformation. Genetic engineering facilitated introduction of desired traits into crops as well as understanding novel gene functions (Anjanappa and Gruissem, 2021; Nerkar et al., 2018a, b; Devarumath et al., 2015). Till date, 525 different transgenic events across 32 crops have been approved for cultivation (Kumar et al., 2020; Anjanappa and Gruissem, 2021). However, the major challenges for plant transformation are the expensive, time-consuming, and recalcitrant crops. Genetic transformation involves random integration of transgenes in the nuclear genome often leading to transgene silencing which can be overcome by precision genome editing.

## Precision Genome Editing for Developing Crops With Important Agronomic Traits

Genome editing has revolutionized the crop improvement through the generation of precise changes in the plant genome that was a long-standing goal of the plant breeders across the globe. Since the first report on genome editing in rice (Liu et al., 2012), genome editing has been reported in and array of food crops such as vegetable crops (Cabbage, Carrot, Pumpkin Tomato Potato, Cucumber, Sweet potato, Basil, Cassava, Chilly, Kale, Lettuce, Lactuca sativa) fruit crops (Apple, Banana, Grapefruit, Coconut, Date Palm, Grapes, Lychee, Melon, Orange, Papaya, Pear, Strawberry, Watermelon, Kiwifruit, Blueberry, Citrus) cereal crops (Barley, Rice Wheat, Maize, Oats) legume crops (Chickpea, Cowpea), sugar producing crops (Sugarcane, Sugar beet) spice crops (Pepper, Saffron) as well as other industrial crops (Coffee, Dandelion, *Jatropha curcas*, Millet, Sorghum, Switchgrass) oil crops (canola, flax, oil palm, oilseed rape, soybean and sunflower) as reviewed by Liu et al. (2021). The application of CRISPR-Cas9 for development of crops with important agronomic traits is discussed in the further sections.

## CRISPR-Cas9 Mediated Genome Editing of Crops for Disease Resistance

CRISPR-mediated engineering of plants for disease resistance has been reported in major crops (as reviewed by Zaidi et al., 2020) such as rice, wheat, tomato, banana, citrus, grapes, cassava and cucumber (Table 2). Broad spectrum resistance is an effective strategy for disease management in crops as these loci confer resistance to diverse species or races of pathogen. Zhou et al. (2018) discovered the *bsr-k1* allele in rice and also developed the *bsr-k1* (broad spectrum resistance Kitaake-1) mutant, which confers broad-spectrum resistance against *Magnaporthe oryzae* and *Xanthomonas oryzae* pv *oryzae* without affecting the major agronomic traits. The bacterial blight caused by *Xanthomonas oryzae* pv. *Oryzae* causes significant yield losses in rice. The expression of sucrose transporter genes *SWEET1*, *SWEET3* and *SWEET14* causes disease susceptibility. Oliva et al. (2019) engineered broad-spectrum resistance into the rice line Kitaake and two mega varieties IR64 and Ciherang-Sub1.

**TABLE 2:** Genome editing using CRISPR-Cas system in major crops for disease resistance and abiotic stress tolerance.

Plant	Gene modified	Function	Agronomic trait	Transformation method	References
Disease resistance					
Rice	*Bsr-k1*	Broad spectrum resistance	Broad spectrum resistance	Agrobacterium-mediated transformation	Zhou et al. (2018)
	*OsSWEET11, OsSWEET13, and OsSWEET14*	Susceptibility genes for bacterial blight	Resistance to bacterial blight	Agrobacterium-mediated transformation	Oliva et al. (2019)
Wheat	TaMlo-A1, TaMloB1, and TaMlo-D1	Mildew resistance locus proteins	Resistance to powdery mildew	Biolistic transformation	Wang et al. (2014)
	TaEdr1 (three homologs)	Negative role in powdery mildew resistance	Resistance to powdery mildew	Biolistic transformation	Zhang et al. (2017)
Tomato	Pmr4	Negatively controls the SA-associated defense pathway	Resistance to powdery mildew	Agrobacterium-mediated transformation	Santillan Martinez et al. (2020)
	Jaz2	Major COR/JA-Ile co-receptor in Arabidopsis controlling stomata dynamics during bacterial invasion	Resistance to bacterial speck disease	Agrobacterium-mediated transformation	Ortigosa et al. (2019)
Banana	RGA2, Ced9	Antiapoptosis gene, prevention of fungal-induced cell death and maintenance of organelle homeostasis	Resistance to *Fusarium* wilt	Agrobacterium-mediated transformation	Dale et al. (2017)
Citrus	CsLOB1	Citrus canker disease susceptibility gene	Resistance to citrus canker	Agrobacterium-mediated transformation	Jia et al. (2017); Peng et al. (2017)
Grapes	VvWRKY52	WRKY transcription factor playing a role in biotic stress	Resistance to *B. cinerea*	Agrobacterium-mediated transformation	Wang et al. (2017)
Cassava	nCBP-1, nCBP-2	Novel cap binding proteins from the eIF4E protein family playing an essential role in the initiation of cap-dependent mRNA translation	Resistance to brown streak disease	Agrobacterium-mediated transformation	Gomez et al. (2019)
Cucumber	eIF4E	Eukaryotic translation initiation factor 4E playing role in biotic stress	Resistance to Cucumber vein yellowing virus (Ipomovirus)	Agrobacterium-mediated transformation	Chandrasekaran et al. (2016)
Abiotic stress tolerance					
Rice	OsMYB30	Cold tolerance	Cold tolerance	Agrobacterium-mediated transformation	Zeng et al. (2020)
	OsNAC14	Transcription factor	Drought tolerance	Agrobacterium-mediated transformation	Shim et al. (2018)
	PQT3	Ubiquitin ligase	Salinity tolerance	Agrobacterium-mediated transformation	Alfatih et al. (2020)
	AOX1a, AOX1b, AOX1c, BEL	Breeding stress marker	Multiple stress tolerance	Agrobacterium-mediated transformation	Xu et al. (2015)
	ALS	Acetolactate synthase	Herbicide tolerance	Agrobacterium-mediated transformation	Endo et al. (2016)
Wheat	DREB2, DREB3, ERF3	Dehydration responsive element binding protein	Drought tolerance	PEG-mediated transformation	Kim et al. (2018)
	EPSPS	Synthesis of amino acids (aromatic)	Herbicide tolerance	Biolistic transformation	Arndell et al. (2019)
	INOX, PDS	Inositol oxygenase, Phytoene desaturase	Multiple stress tolerance	Agrobacterium-mediated transformation	Upadhyay et al. (2013)
Maize	ALS	Acetolactate synthase	Herbicide tolerance	Biolistic transformation	Yadava et al. (2017)
Sugarcane	ALS	Acetolactate synthase	Herbicide tolerance	Biolistic transformation	Oz et al. (2021)
Soybean	ALS1	Acetolactate synthase	Herbicide tolerance	Biolistic transformation	Li et al. (2015)
Tomato	BZR1	Brassinosteroid regulator	Heat stress	Agrobacterium-mediated transformation	Yin et al. (2018)
	NPR1	Drought tolerance	Drought tolerance	Agrobacterium-mediated transformation	Wang et al. (2015); Li et al. (2019)
	CLV3	Regulates shoot and Floral meristem development	Salinity stress tolerance	Agrobacterium-mediated transformation	Li et al. (2018); Van Eck et al. (2019)
	PDS	Carotenoid biosynthesis	Multiple stress tolerance	Agrobacterium-mediated transformation	Woo et al., 2015
	ALS	Acetolactate synthase	Herbicide tolerance	Biolistic transformation	Veillet et al. (2019a)
*Brassica* napus	BnaA6.RGA (DELLA Protein)	Transcription factor	Drought tolerance	Agrobacterium-mediated transformation	Wu et al. (2020)

Simultaneous mutation of the three homeoalleles of TaMLO conferred heritable broad-spectrum resistance to powdery mildew in hexaploid bread wheat (Wang et al., 2014). Similarly, Zhang et al. (2017) generated wheat *edr1* plants by simultaneous modification of the three homoeologs of TaEDR1 confirming its negative role in powdery mildew resistance. In tomato, Powdery Mildew Resistance 4 *PMR4* knock-out lines showed enhanced resistance against powdery mildew pathogen *Oidium neolycopersici* (Santillan Martinez et al., 2020). Genetic manipulation of defence pathways is limited due to the antagonistic interactions between the SA and JA defence pathways. Ortigosa et al. (2019) reported spatial uncoupling the SA-JA antagonism at the stomata and generated a tomato resistant to the bacterial speck disease caused by the pathogen Pto DC3000, without compromising resistance to necrotrophic pathogens, by editing the SlJAZ2 gene (Santillan Martinez et al., 2020). Resistance to *Fusarium* wilt (Banana), citrus canker (Citrus), *B. cinerea* (grapes), brown streak disease (Cassava) and *Ipomovirus* (cucumber) has also been reported (Dale et al., 2017; Jia et al., 2017; Peng et al., 2017; Wang et al., 2017; Gomez et al., 2019; Chandrasekaran et al., 2016; Table 2).

## Development of Abiotic Stress Tolerant and High-Yielding Crops Using CRISPR-Cas9

Abiotic stresses pose a major threat to the crop yield and productivity. CRISPR/Cas has been adopted rapidly for the manipulation of crop genomes to develop abiotic stress tolerant and high-yielding mutants (Bhat et al., 2021). Simultaneous editing of three genes, OsPIN5b (a panicle length gene), GS3 (a grain size gene) and OsMYB30 (a cold tolerance gene) with the CRISPR-Cas9 resulted in several new rice mutants with high yield and excellent cold tolerance (Zeng et al., 2020; Table 2) which was also stable in the T2 generation. Overexpression of the BZR (brassinosteroid regulator) in tomato conferred thermo-tolerance via regulation of the Feronia (Fer) homologs (Yin et al., 2018).

Improvement in drought tolerance by modulating the important transcription factors has been reported in the major crops like rice (Shim et al., 2018), wheat (Kim et al., 2018), tomato (Wang et al., 2015; Li et al., 2019) and *Brassica napus* (Wu et al., 2020). Shim et al. (2018) reported the functional characterization of the rice drought responsive transcription factor OsNAC14. Overexpression of OsNAC14 conferred drought tolerance in the rice mutants at the vegetative stage of growth. Field performance of OsNAC14 overexpressing transgenic rice lines revealed that these lines exhibited higher number of panicle and filling rate compared to non-transgenic plants under drought conditions. In wheat, CRISPR-Cas9 mediated genome editing of dehydration responsive element binding protein 2 (TaDREB2) and ethylene responsive factor 3 (TaERF3) resulted in improved drought tolerance (Kim et al., 2018). Li et al. (2019) isolated *SlNPR1* (non-expressor of pathogenesis-related gene 1) from tomato and generated *slnpr1* mutants using the CRISPR/Cas9 system and found that lines overexpressing *SlNPR1* showed reduced drought tolerance. This work throws a light on function of NPR1 in plant response. In rapeseed, CRISPR-Cas9 editing of *bnaa6. rga-D* and *bnarga* genes helped in understanding roles of DELLA proteins in drought tolerance in *B. napus* (Wu et al., 2020). The *bnaa6. rga-D* mutants displayed enhanced drought tolerance and *BnaRGA*s physically interacted with BnaA10. ABF2, an essential transcription factor in ABA signaling.

Major work on salinity tolerance has been done in rice (as reviewed by Bhat et al. (2021). The Arabidopsis PARAQUAT TOLERANCE 3 (*AtPQT3*) encoding an E3 ubiquitin ligase confers an off-switch mechanism which enable plants to balance the growth and stress responses (Alfatih et al., 2020). *OsPQT3*, a rice homologue of *AtPQT3* was knock-out using CRISPR-Cas9 (Alfatih et al., 2020). The resulting *OsPQT3* knockout mutants (*ospqt3*) displayed enhanced resistance to oxidative and salt stress significantly enhanced agronomic performance with higher yield compared with the wild type under salt stress in greenhouse and in field conditions. Li et al. (2018) introduced desirable traits into four stress-tolerant wild-tomato accessions by using multiplex CRISPR–Cas9 editing of genes associated with morphology, flower and fruit production, and ascorbic acid synthesis. The Cas9-free progeny of edited plants had domesticated phenotypes and also retained disease resistance and salt tolerance traits from the parents (Li et al., 2018).

Herbicide tolerance has been engineered in rice (Endo et al., 2016), maize (Yadava et al., 2017), sugarcane (Oz et al., 2021), soybean (Li et al., 2015) and tomato (Veillet et al., 2019b) by editing the Acetolactate synthase (*ALS*) gene; and in wheat (Arndell et al., 2019) by editing the 5-enolpyruvylshikimate 3-phosphate synthase (*EPSPS*) gene to develop crop varieties resistant to chlorsulfuron and glyphosate, respectively.

In rice, CRISPR-Cas9 mediated gene editing of GS3 and Gn1a genes responsible for grain size and grain number resulted into generation of 3 mutant genotypes (gs3-N9108, gs3-Z22, and gs3gn1a-Z22) which showed 3–7% increase in grain yields than the WT (Shen et al., 2018). Hao et al. (2019) reported larger grain size in the genome edited mutants generated by editing GL2/OsGRF4 and OsGRF3 genes responsible for grain size and grain yield, respectively. CRISPR-Cas9 mediated genome editing of Gn1a, DEP1, GS3, IPA1 led to enhanced grain number; dense erect panicles; larger grain size; and variation in the tiller number in T2 generation (Li et al., 2016).

## Biofortification of Crops Using CRISPR-Cas9

Biofortification of grains is one of the main goals of breeders to enhance the nutritive value of grains for controlling the nutrient-deficiency related diseases. Lysine content has been improved by up to 25-fold in rice by editing the gene *AK* (*lysC*) and *DHPS* (*dapA*) responsible for key enzymes in lysine biosynthesis (Yang et al., 2016) (Table 3). In addition, these high-lysine lines showed improved physic-chemical properties without affecting the starch composition. The plants showed normal growth in field trials with slight difference in plant height and grain colour (Yang et al., 2016). Carotenoid biofortification has been achieved in rice by genome editing of CrtI and PSY genes resulting in marker-free gene-edited mutants containing high β-carotene content (Dong et al., 2020). Biofortified tomato has been produced with diverse nutrient like γ-aminobutyric acid (GABA). GABA is a neurotransmitter that control anxiety and blood pressure. By deleting the C-terminal autoinhibitory domain of glutamate decarboxylase, a key enzyme in GABA biosynthesis, mutant tomatoes have been created in which GABA accumulation increased by seven-fold (Nonaka et al., 2017). Yellow-seeded mutants in rapeseed have been created using the CRISPR-Cas9 mediated editing of BnTT8 homologs which increased the oil content in the T2 generation by 9.47%. These BnTT8 double mutants with high oil yield potential and modified FA composition as well as improved the nutritional quality could have potential application in rapeseed breeding (Zhai et al., 2020). Tuncel et al. (2019) investigated Cas9-mediated mutagenesis of starch-branching enzymes (SBEs) in tetraploid potatoes and developed transgene-free mutant potato lines with elevated levels of resistant starch which can help in improving insulin control of blood sugar levels. Taken together, these results demonstrate that Cas9-mediated mutagenesis holds promise for development of commercially viable crops.

**TABLE 3:** Genome editing using CRISPR-Cas system in major crops for increased yield, improved nutritive value, reduction in anti-nutritional factors and improved shelf-life.

Plant	Gene modified	Function	Agronomic trait	Transformation method		References
Increased yield						
Rice	GS3 and Gn1a	GS3: QTL regulating grain size; Gn1a: QTL regulating grain number	Grain size and grain number		Agrobacterium-mediated transformation	Shen et al. (2018)
	GL2/OsGRF4 and OsGRF3	GL2 transcript negatively regulated grain size and yield	Grain size and yield		Agrobacterium-mediated transformation	Hao et al. (2019)
	Gn1a; DEP1; GS3; IPA1	Gn1a: regulates grain number; DEP1: regulates panicle size; GS3: regulates grain size; IPA1: regulates plant architecture	grain number; panicle architecture; grain size; plant architecture		Agrobacterium-mediated transformation	Li et al. (2016)
Improved nutritive value						
Rice	AK (lysC) and DHPS (dapA)	Key enzymes in lysine biosynthesis	Lysine content		Agrobacterium-mediated transformation	Yang et al. (2016)
	CrtI, PSY	Carotenoid biosynthesis	High β-carotene content		Agrobacterium-mediated transformation	Dong et al. (2020)
Tomato	SlGAD2, SlGAD3	Glutamate decarboxylase- key enzyme in GABA synthesis	High GABA content		Agrobacterium-mediated transformation	Nonaka et al. (2017)
Potato	StSBE1, StSBE2	Starch branching enzymes	High amylose content		Agrobacterium-mediated transformation/PEG -mediated transformation	Tuncel et al. (2019)
Rapeseed	BnTT8	Transcription factor regulator activating pro anthocyanidins-specific genes in seed coat development	High oil production and GPC		Agrobacterium-mediated transformation	Zhai et al. (2020)
Reduction in anti-nutritional factors						
Rice	OsNramp5	Cd transporter mediating root uptake of Cd	Cd accumulation		Agrobacterium-mediated transformation	Tang et al. (2017)
	OsPLDα1	Regulates abscicic acid signalling	Low phytic acid content		Agrobacterium-mediated transformation	Khan et al. (2019)
Wheat	α-gliadin genes	Gluten proteins	Low gluten content		Biolistic transformation	Sanchez-Leon et al. (2018)
Rapeseed	BnITPK	Key enzyme ITPK (inositol tetrakisphosphate kinase), catalysing the penultimate step for the synthesis of Phytic Acid in plants	Low phytic acid content		Agrobacterium-mediated transformation	Sashidhar et al. (2020)
Improved shelf-life						
Tomato	ALC	An allele of nor (non-ripening) gene	Extended shelf life		Agrobacterium-mediated transformation	Yu et al. (2017)
	PL, PG2a, TBG4	Tomato pectin degrading enzymes determining softening in fleshy fruits	Long shelf life		Agrobacterium-mediated transformation	Wang et al. (2019)
	RIN	MADS-box transcription factor regulating fruit ripening	Slower ripening		Agrobacterium-mediated transformation	Ito et al. (2015)
Banana	MaACO1	Encodes ACC oxidase playing a role in ripening	Long shelf life		Agrobacterium-mediated transformation	Hu et al. (2020)
Petunia	PhACO1	Encodes ACC oxidase and expressed during flower development	Increased shelf life		PEG-mediated transfection	Xu et al. (2020)

## Reduction in Anti-Nutritional Factors in CRISPR-Cas9 Edited Crops

In order to reduce the phytic acid content in rapeseeds, the *ITPK* gene encodes an enzyme that catalyzes the penultimate step of phytate synthesis. In rapeseed, the *ITPK* gene knock-out by CRISPR/Cas9 led to reduction in the phytic acid content by 35% (Table 3) without affecting the plant performance (Sashidhar et al., 2020). The gluten protein in wheat is another important anti-nutritional factor which can cause coeliac disease in gluten intolerant individuals. Reduction of the gluten content using the conventional breeding methods is difficult as this protein is encoded by more than 100 loci in the wheat genome. CRISPR/Cas9 mediated targeting of a conserved region of the α-gliadin genes has led to the production of low-gluten and transgene-free wheat lines (Sanchez-Leon et al., 2018). A remarkable application of the CRISPR/Cas9 technology in rice breeding is the generation of heavy metal pollution-safe rice cultivars. Cadmium (Cd) is a human carcinogen which can also lead to renal failure upon long-term consumption. Tang et al. (2017) developed novel Indica rice cultivars accumulating low Cd levels in the grain by mutating the *OsNramp5* gene, which mediates the root uptake of Cd. Field performance evaluation of osnramp5 mutants revealed that high Cd conditions did not affect the agronomic traits and the grain yield (Tang et al., 2017; Liu et al., 2021).

## Genome Editing for Developing Resilient Crops in Changing Climatic Conditions

**FIGURE 2:**
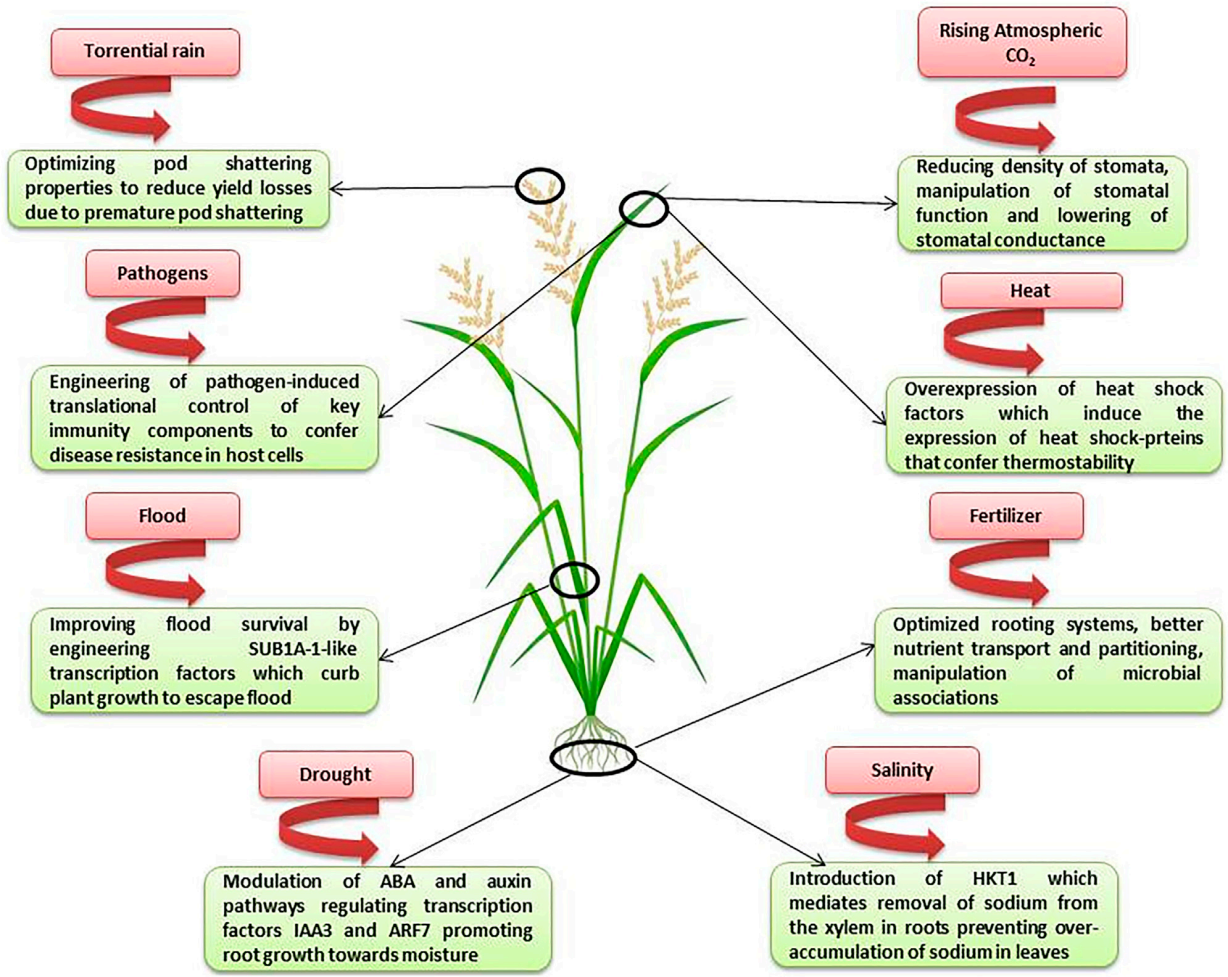
Genome editing to develop high-yielding and climate resilient crops (Abbreviations: SUB1A-1 Submergence 1A (SUB1A) which confers tolerance by quiescence of growth; ARF7 (auxin response factor); HKT1 (high-affinity K+ transporter sub-family 1) which mediates sodium (Na+) exclusion from leaves).

Genome editing tools have become the most widely used biotechnological tools in crop breeding. Presently, the genome editing of crops is at a stage of elucidating the genomic function and regulatory mechanisms (Liu et al., 2021) and there is a long way to go before the translation of research on genome edited crops from lab to the field. The climate change continues to be the major limiting factor in the crop improvement. Therefore, increasing crop yield in the sub-optimal environments is the most important goal for the breeders. Bailey-Serres et al. (2019) have enlisted the different factors affecting the crop productivity and suggested the breeding strategies for increasing crop yield in sub-optimal environments. Genome editing indeed has a crucial role to play in elucidating the gene functions during stress responses as well as the adaptive mechanisms that plants have evolved in response to the harsh environmental conditions. Figure 2 depicts the areas where genome editing can find applications in breeding high-yielding and climate-resilient crops.

## Regulatory Approval of the Genome-Edited Crops

Genome editing is an innovative plant breeding technological advancement which creates targeted changes in the plant’s own genome without the insertion of transgenic sequences. Genome editing is also referred to as a New Breeding Technique. Researchers argue that genome editing makes small genetic changes that could be found in nature. This is clearly different from introducing the DNA from other species into plant genomes. Unlike the older approaches, gene editing allows researchers to make more targeted changes in the genome.

Regulation of the genome-edited crops is crucial for its applicability for the betterment of crops which provide food, fibre and fuel for the growing population of the world in the face of a global climate change. On one hand when this technology has proved its versatile application in an array of important crops like rice, wheat, corn, soybean, tomato, potato, banana, cassava and oranges; international discussions are seeking legal clarity about the regulatory approval of genome editing and derived products (Lassoued et al., 2021). In 2019, soybean variety producing oil with a longer shelf life became the first commercialized gene edited food product to be launched in the United States by Calyxt of Roseville, Minnesota. Earlier this year, a gene-edited tomato with higher amounts of γ-aminobutyric acid (GABA) came in to the market in Japan. Recently, United Kingdom has planned to ease requirements for field research on gene-edited crops by allowing the researchers to conduct field trials of gene-edited plants without the need to submit risk assessments (Ledford 2021). Recognizing that the SDN1 (that introduce changes in the plant genome through small insertions/deletions) and SDN2 (that uses a small template to generate a desired change in the plant genome) categories of plants are free from any transgenes, the Ministry of Environment, Forest and Climate Change (Government of India) issued a notification on 30 March 2022 to exempt products of SDN1 and SDN2 (free from transgenes) from the provisions of Rules 7 and 11 (both inclusive) of Rules, 1989, whereas products of SDN3 (with transgenes) will be treated in the same way as GE organisms under Rules, 1989 (Ahuja, 2022). This decision will further boost the research and development of genome-edited products in India. However, genome-edited plants still need regulatory exemption from most of the countries in the world. Nevertheless, this will further boost the research and development of genome edited crops in India. However, as the many countries across the still await the exemption of genome-edited products from regulation, scientists believe that genome editing encompasses powerful tools for future food security that should be enabled and not delayed.

Any mutations leading to obviously deleterious phenotypes would be eliminated from breeding programmes (Carroll et al., 2016). Other hypothetical risks, such as a modified protein that turned out to be allergenic to humans, might equally well arise naturally in the absence of human intervention. The effects of genome editing are largely identical to those of the natural processes that continually create variation in the genomes of food animals. From this point of view, it is hard to see why the process of genome editing to introduce defined genetic changes should be regulated when the process of spontaneous mutation that introduces new random changes into every individual’s genome, every generation, is not. Genome editing allows precise changes to be made in the genomes of agricultural organisms without the introduction of DNA from other species. The products of editing should be subject to the same oversight as other food products, based on the result rather than the process that yielded the result. This technology was developed largely with public funding, and the public should benefit from its intelligent and careful application.

Despite their promise, it is clear that not every issue can or should be solved with these technologies; many are societal problems that must be addressed by changing behavior and mindsets. Decisions to use, not to use, or how to use these tools should be made by informed stakeholders-including consumers and famers in collaboration with plant breeders. Using crops created through genetic engineering and genome editing cannot replace sustainable practices, such as cover cropping, crop rotation, or crop diversification. They can ideally be used in concert with these practices, serving as one tool of the many, that farmers at all production levels can use to adjust to local conditions and challenges.

## Conclusion

Advances in the breeding strategies through the application of innovative technologies have the potential to furnish solutions to address the future challenges in global food security. Combining genetic resources and innovative technologies like genome editing is important for developing crops with important agronomic traits that not only increase the global food security but also reduce the effects of agriculture on the environment. In less than a decade, CRISPR/Cas9 system has become the most widely used tool crop breeding. Considerable progress has been made in developing disease resistant and abiotic stress tolerant crops with improved yield, nutritive value and increased shelf life. Understanding novel gene functions and the regulatory mechanisms of genes controlling important agronomic traits in plants shall facilitate further progress in the application of the genome editing technologies for crop improvement. Through the identification and editing of genes involved in stress tolerance and yield improvement, it would be possible to develop robust crops that are resilient to the global climate change. Although, the translation of genome-edited crop research to the field is still a far way to go, the regulatory approval and consumer acceptance will play an essential role in commercializing the existing genome-edited crops.
